# Modification of the Physical Properties of a Nafion Film Due to Inclusion of n-Dodecyltriethylammonium Cation: Time Effect

**DOI:** 10.3390/polym15112527

**Published:** 2023-05-30

**Authors:** Javier Zamudio-García, María V. Martínez de Yuso, Ana L. Cuevas, David Marrero-López, Juana Benavente

**Affiliations:** 1 Departamento de Química Inorgánica, Cristalografía y Mineralogía, Universidad de Málaga, 29071 Málaga, Spain; zamudio@uma.es; 2 Servicios Centrales de Investigación, Universidad de Málaga, 29071 Málaga, Spain; mvyuso@uma.es; 3 Unidad de Nanotecnología, Centro de Supercomputación y Bioinnovación, Servicios Centrales de Investigación, Universidad de Málaga, 29071 Málaga, Spain; analaura.cuevas@uma.es; 4 Departamento de Física Aplicada I, Universidad de Málaga, 29071 Málaga, Spain

**Keywords:** Nafion, dodecyltriethylammonium cation, electrical resistance, optical properties, elastic modules

## Abstract

This study investigates the effects of modifying commercial Nafion-212 thin films with dodecyltriethylammonium cation (DTA+) on their electrical resistance, elastic modulus, light transmission/reflection and photoluminescence properties. The films were modified through a proton/cation exchange process for immersion periods ranging from 1 to 40 h. X-ray diffraction (XRD) and X-ray photoelectron spectroscopy (XPS) were employed to analyze the crystal structure and surface composition of the modified films. The electrical resistance and the different resistive contributions were determined via impedance spectroscopy. Changes in the elastic modulus were evaluated using stress–strain curves. Additionally, optical characterization tests, including light/reflection (250–2000 nm) and photoluminescence spectra, were also performed on both unmodified and DTA^+^-modified Nafion films. The results reveal significant changes in the electrical, mechanical and optical properties of the films, depending on the exchange process time. In particular, the inclusion of the DTA^+^ into the Nafion structure improved the elastic behavior of the films by significantly decreasing the Young modulus. Furthermore, the photoluminescence of the Nafion films was also enhanced. These findings can be used to optimize the exchange process time to achieve specific desired properties.

## 1. Introduction

Nafion is a well-known family of polymers that was discovered in the late 1960s by DuPont. This comprises a tetrafluoroethylene (PTFE) backbone into which perfluorovinyl ether chains ended by sulfonic groups are incorporated [1]. Thus, Nafion combines the high hydrophobicity of the polytetrafluoroethylene backbone with the great hydrophilicity of the sulfonic groups placed at intervals along the chain. These acid groups, which are known to aggregate in clusters, allow the transport of ions and serve as a polymer electrolyte [2,3]. In fact, Nafion is the most commonly used proton conductor electrolyte in proton exchange membrane fuel cells (PEMFCs) [4,5,6,7]. In addition, the mechanical and thermal stability of Nafion membranes (or thin films), as well as their optical and photoluminescence properties, are also of great interest for a variety of applications [8,9,10,11,12]. Modifications of Nafion membranes have been carried out to improve their mechanical and chemical stability properties at temperatures higher than 80 °C. In particular, the modification of Nafion films via the incorporation of different nanoparticles, such as oxides (SiO_2_ and TiO_2_) or metals (Ag, Pt, and Cd), have been reported [13,14,15,16,17]. In this context, the unique chemical nanostructure of Nafion, characterized by segregated hydrophilic and hydrophobic domains, enables its use as a template for the synthesis of metal nanoparticles inside its channels [18,19]. This process not only enhances the catalytic activity of the resulting nanocomposites, but also increases their mechanical stability. On the other hand, the inclusion of different room-temperature ionic liquids (or ionic liquids cations) leads to a significant reduction in the water loss or in the methanol crossover, being a point of interest in the case of direct methanol fuel cells [6,20].

Room-temperature ionic liquids (RTILs or ILs) are molten salts with a very low vapor pressure comprising an organic cation and an inorganic/organic anion, which remain in a liquid state at temperatures below 100 °C. Furthermore, they also exhibit high solubility and conductivity, thermal stability and water sensitivity [21]. As a result, they have gained significant attention as environmental alternatives to volatile organic compounds since the mid-1970s. Additionally, their elevated viscosity makes them suitable for a wide range of applications in different industrial and research areas, such as electrodeposition, extraction, energy, conducting membranes, electrochemical devices or optical sensors [22,23,24]). On the other hand, the possibility of tailoring the most adequate physicochemical properties in order to specifically apply the LIs via the selection of the cation/anion is another interesting characteristic. Nevertheless, the liquid state of ILs can sometimes be a disadvantage when integrating them into devices, requiring immobilization through inclusion or deposition within solid structures or films (either inorganic or polymeric). In this context, the deposition of ILs on alumina nanostructures has been demonstrated to enhance the efficiency of solar cells [24], while IL/biopolymer hybrid materials are being developed for biomedical applications [25,26].

The aim of this study is to investigate the changes in the various physical properties of a commercial Nafion thin film, including its electrical resistance, elastic modulus, light transmission/reflection and photoluminescence, after the inclusion of the dodecyltriethylammonium cation (DTA^+^) via a proton/cation exchange process. The degree of cation inclusion was varied by changing the exchange times (t = 1, 2, 8, 22 and 40 h), and both X-ray diffraction (XRD) and X-ray photoelectron spectroscopy (XPS) techniques were used to analyze the time evolution of DTA^+^ into the structure of the films. The modification of the electrical resistance was determined using impedance spectroscopy measurements, while stress–strain curves were used to determine changes in the elastic modulus. Light transmission/reflection measurements for wavelengths from 250 to 2000 nm, and photoluminescence spectra were obtained for modified Nafion films. These measurements confirmed that each physical property undergoes different modifications during the exchange process, demonstrating the ability to tailor these properties to specific requirements.

## 2. Materials and Methods

### 2.1. Material Preparation

A Nafion 212 film (DuPont, Wilmington, DE, USA) was selected for modification with n-dodecyltriethylammonium chloride (DTA^+^Cl^−^) (chemical formulae C_12_H_25_N(CH_3_)_3_Cl, Merck, Darmstadt, Germany) via a proton/cation exchange process [27], similar to that previously reported for a Nafion 112 membrane and different ILs cations [13,28,29]. The DTA^+^ molecular weight, molar volume and density (20 °C) were 228 g mol^−1^, 350 cm^3^ mol^−1^ and 870 kg m^−3^, respectively.

Different portions of the Nafion film were immersed in a 40% (*w*/*w*) DTA^+^Cl^−^ aqueous solution for different periods of time, t = 1, 2, 8, 22 and 40 h. Nafion film modification was performed by Dr. L. Neves (REQUIMTE/CQFB, Departamento de Química, Universidad Nova de Lisboa, Caparica, Portugal). These samples are hereafter denoted as Naf/DTA^+^ (1 h), Naf/DTA^+^ (2 h), Naf/DTA^+^ (8 h), Naf/DTA^+^ (22 h) and Naf/DTA^+^ (40 h). Prior to the different characterization measurements, the films were stored in air atmosphere for several weeks.

The films’ thickness, measured using a Coolant Proof Micrometer (mod. IP65, Mitutoyo, Kanagawa, Japan), increased after the DTA^+^ inclusion from 50 µm for the untreated Nafion film to 58 µm for Naf/DTA^+^ (40 h).

### 2.2. Structural and Chemical Surface Characterization

X-ray diffraction patterns were collected using a PANalytical Empyrean X-ray diffractometer and CuKα radiation in the 2θ range of 5–80°, with a total acquisition time of 1 h. Phase identification and analysis were performed using X’Pert HighScore Plus software v. 5.2.

The FTIR spectra of the Nafion films were recorded by using a Perkin Elmer Spectrum 100.

The chemical surface composition of the films was determined using X-ray photoelectron spectroscopy (XPS) with a Physical Electronics spectrometer (PHI 5700) and X-ray Mg K_α_ radiation (300 W, 15 kV and 1253.6 eV). High-resolution spectra were recorded via a concentric hemispherical analyzer operating in the constant pass energy mode at 29.35 eV, using a 720 µm diameter analysis area at the optimum equipment take-off angle (45°). The films were kept overnight under high vacuum (preparation chamber) and then transferred to the analysis chamber for testing. The residual pressure in the analysis chamber during the data acquisition was maintained below 5 × 10^−7^ Pa. Each spectral region was scanned several times to obtain a good signal-to-noise ratio. The binding energies were determined with respect to the position of the adventitious C 1s peak at 285.0 eV (accurate ± 0.1 eV). The acquisition and data analysis were performed using the PHI ACCESS ESCA-V6.0 F software package. The atomic concentration percentages (A.C.%) of the characteristic elements were determined taking into account the corresponding area sensitivity factor for each measured spectral region. The spectra of the different element core levels were fitted using Gauss–Lorentz curves [30]. Unfortunately, XPS measurements could not be performed for the Naf/DTA^+^ (40 h) sample due to a vacuum loss during analysis.

### 2.3. Electrical Characterization

The electrical resistance of the Nafion films was determined via impedance spectroscopy measurements using a frequency response analyzer (FRA, Solartron 1260, Hampshire, UK) for frequencies ranging between 0.01 and 10^6^ Hz with an ac voltage of 50 mV. The impedance spectra were collected at room temperature and a relative humidity of 60% by pressing the samples between two porous carbon electrodes (Sigracet, GDL 10 BB, Texas, USA). The impedance spectra were analyzed using equivalent circuit models and Distribution of Relaxation Times (DRT) using the ZView (Scribner Associates Inc., Southern Pines, NC, USA) and DRTtools software DRTtools (1.0, Matlab 7.2) [31], respectively.

### 2.4. Elastic Characterization

The elastic properties of the films were measured using a Mark-10 Tensile Tester, equipped with a digital force gauge (ES20 model) and controlled with MESUR gauge software v.1.8.2 (Copiague, NY, USA). The tensile strength curves were collected for rectangular samples of 3 × 1 cm^2^ at a strength rate of 1 cm·min^−1^ at ambient conditions. The elastic or Young modulus (Y) was determined from the slope of the initial linear region of the stress–strain curves. Two different samples were tested in order to obtain the stress–elongation curves, and the resulting average elastic modulus values are provided.

### 2.5. Optical Characterization

The optical characterization was performed by analyzing the light transmittance/reflection and photoluminescence (PL) spectra. Transmittance/reflection measurements were performed using a Varian Cary 5000 spectrophotometer (Agilent Technologies, Santa Clara, CA, USA) equipped with an integrating sphere of Spectralon in the wavelength range of 250 to 2000 nm. Photoluminescence (PL) spectra at room temperature were measured using a HORIBA Scientific LabRam PL Microscope, which was equipped with a laser as the excitation light source. The laser had a wavelength range of 325 to 750 nm and a beam power of 0.28 mW.

## 3. Results

### 3.1. XRD and XPS Characterization

**Figure 1:**
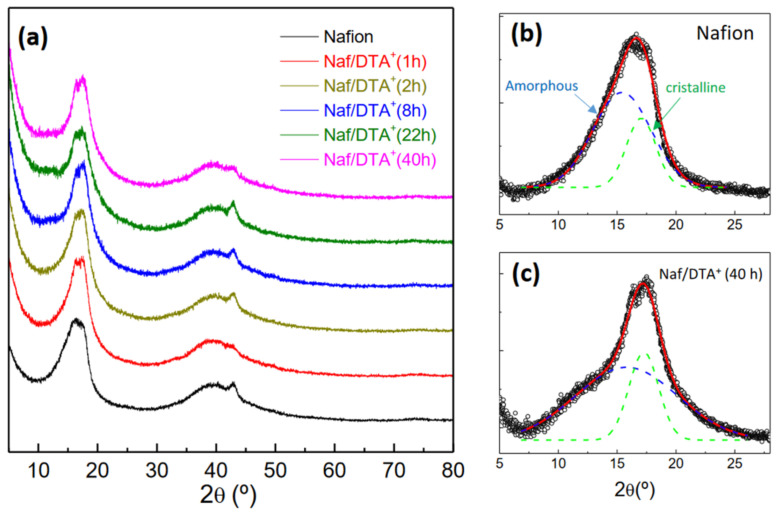
(**a**) XRD patterns of Nafion and DTA^+^-modified Nafion films at different exchange times. Details of the (100) reflection for Nafion (**b**) and Naf/DTA^+^ (40 h) (**c**), showing the amorphous and crystalline components.

Nafion films are known to have a certain degree of crystallinity in their crystal structure, which is critical for their structural integrity and mechanical stability [32]. The XRD patterns, shown in Figure 1a, present two broad diffraction peaks at 2θ ~17 and 39°, which can be attributed to the semicrystalline nature of the perfluorocarbon peak chains of the ionomer. Moreover, no significant changes in the position of the characteristic peaks after DTA^+^ modification are observed. A closer inspection of the patterns reveals that the unmodified Nafion films exhibit two overlapped peaks in the 2θ range of 15–20° (Figure 1b). One of these peaks corresponds to the amorphous component near 15°, with a width of half maximum (FWHM) of 5.6°, while the other corresponds to the crystalline phase near 17°, with a FWHM of 2.5°. This peak is assigned to the (100) reflection of the hexagonal crystal structure of Nafion [32]. The DTA^+^-modified Nafion films display noticeable changes in the peak corresponding to the amorphous component (Figure 1c). Specifically, the fraction of the crystalline phase slightly decreases after DTA incorporation, from 27 wt.% for the unmodified dry film to 23 wt.% for the Naf/DTA (40 h) sample. Thus, the H^+^/DTA^+^ exchange does not alter significantly the crystalline regions, suggesting a reorganization of the amorphous structure. Similar findings were previously reported for lithiated Nafion membranes [33].

The incorporation of DTA^+^ into Nafion membranes was further studied by the FTIR (Figure S1, Supplementary Information). The transmittance spectrum of the pristine Nafion film exhibited characteristic bands at 1210 and 1140 cm^−1^ (asymmetric and symmetric stretching of CF_2_ groups, respectively), 1050 cm^−1^ (symmetric stretching of SO_3_ groups), and two bands at 980 and 970 cm^−1^ (symmetric stretching of C–O–C). Additionally, a doublet band at about 2350 cm^−1^ was observed due to atmospheric carbon dioxide [34,35,36]. After DTA+ modification, the position and intensity of the characteristic peaks of Nafion remained unchanged, but new bands were clearly discernible at 2930 and 2850 cm^−1^ (asymmetric and symmetric stretching of CH_3_ and CH_2_ aliphatic groups, respectively) and 1490 cm^−1^ (bending of aliphatic groups). Interestingly, the relative intensity of these bands increased after DTA^+^ modification, confirming the progressive incorporation of the cation into the films with increasing time.

**Figure 2:**
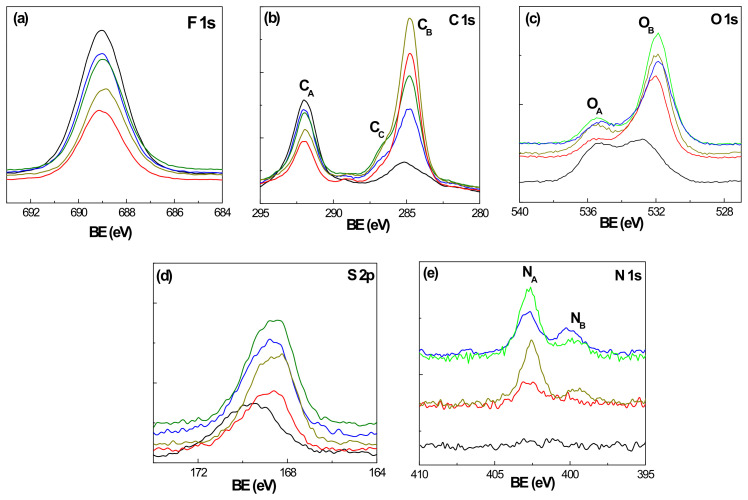
Core-level spectra of the elements detected on the surface of the films: fluorine (**a**), carbon (**b**), oxygen (**c**), sulfur (**d**) and nitrogen (**e**). Nafion (black line), Naf/DTA^+^ (1 h) (red line), Naf/DTA^+^ (2 h) (dark-yellow line), Naf/DTA^+^ (8 h) (green line) and Naf/DTA^+^ (22 h) (blue line).

The XPS technique was employed to determine the chemical surface composition of the Nafion and Naf/DTA^+^ (t) films. The high-resolution spectra of the Nafion characteristic elements (C, F, O and S), as well as nitrogen associated with DTA^+^ incorporation, were analyzed (Figure 2). The F 1s core-level spectra (Figure 2a) show a peak of 689.0 eV at practically the same binding energy for all samples (BE) (C–F_2_ link [30]). However, the C 1s spectra (Figure 2b) show two main peaks, one at a BE of 292.0 eV (C_A_), attributed to CF_2_ and CF–O links, and the other at 284.8–285.0 eV (C_B_), assigned to a C–H bond (aliphatic carbon). A small shoulder (C_C_) at 286.7 eV (C–N link) is also detected for modified Naf/DTA^+^ (t) films. Additionally, two shoulders are observed at 293.6 eV (C–F_3_ link) and 289.4 eV (O–C=O link) [37], with a similar area for the Nafion film (~7.5%), but reduced in the case of the modified Naf/DTA^+^ (t) films. The O 1s core-level spectra in Figure 2c show two contributions for the different films at 535.3 eV (O_A_), attributed to the CF–O link of the Nafion structure, and the other at 532.7 eV (O_B_), attributable to the SO_3_ link [30]. This latter contribution shifts to lower BE (531.8 eV) in the case of the modified Naf/DTA^+^ (t) films due to changes in its chemical environment due to the H^+^/DTA^+^ exchange process. The S 2p core-level spectrum for the Nafion film (Figure 2d) displays a broad and symmetric peak at around 169.6 eV. The inclusion of DTA^+^ causes a slight modification in the shape of the curve, shifting the peak maximum to lower BE values (168.2 eV), which is consistent with the behavior observed for the O1s spectra after H^+^/DTA+ exchange. Figure 2e presents the evolution of the N 1s contribution over the exchange time, where a peak assigned to the protonated nitrogen of DTA^+^ is observed at 402.5 eV (N_A_). Another peak attributed to the hydration of matrix impurities at around 400.0 eV (N_B_) is also detected, and its intensity increases with the immersion time [38].

The atomic concentration percentages (A.C.%) of the different elements detected on the surface of the Nafion and Nafion-DTA^+^ (t) films were obtained by analyzing the corresponding core-level spectrum curves shown in Figure 2, and are presented in Table 1. As expected, the inclusion of DTA^+^ results in a reduction in the percentage of fluorine (around 40% after 1 or 2 h immersion time, but around 25% for the longer periods analyzed), and an increase in the contribution of carbon (~38% and 15%, respectively), indicating the progressive inclusion of DTA^+^ into the Nafion structure. The Naf/DTA^+^ (t) samples also exhibit a higher nitrogen and oxygen content, as a result of the aqueous-DTA^+^ solution. The slight increase in sulfur is attributed to the reorganization of surface polymer chains. Additionally, the Nafion-DTA^+^ (t) films show a small percentage of silicon (<4%), but no chlorine was detected, indicating the adequacy of the proton/cation exchange process.

Further analysis was conducted by deconvoluting the core-level spectra of carbon, oxygen and nitrogen, and the corresponding contributions of the different peaks are summarized in Table 2. The A.C.% for C_A_ and C_B_ contributions (C–F_2_ and C–H/C–C links, respectively) clearly show the chemical changes in the film surface as a result of the gradual inclusion of DTA^+^. The significant initial increase in the C–H/C–C link percentage (4 times) is attributed to the surface deposition of DTA^+^ and the corresponding reduction in the C–F_2_ percentage (around 50%), as well as their subsequent decrease/increase due to the inclusion of DTA^+^ in the bulk film structure.

### 3.2. Physicochemical and Electrical Characterization of the Thin Films

Electrochemical impedance spectroscopy (EIS) measurements are commonly used for the electrical characterization of both homogeneous and non-homogeneous materials. This is achieved by analyzing the Nyquist plots, which show the relationship between the real (Z_real_) and imaginary (Z_img_) parts of the impedance data. These parameters are directly related to the electrical resistance and capacitance of the samples, respectively. Moreover, qualitative information of interest regarding the effect of modifications on both the bulk sample and electrode/sample interface can also be obtained [39,40,41,42]. In the case of homogeneous material, the Nyquist plot is a single semicircle with a center in the real axis, which can be simulated by using a resistance and a capacitance in parallel. For non-homogeneous materials, the Nyquist plots typically show depressed semicircles and can be described by (RQ) elements, where R is a resistance in parallel with a non-ideal capacitance or a constant phase element Q(ω) [39].

**Figure 3:**
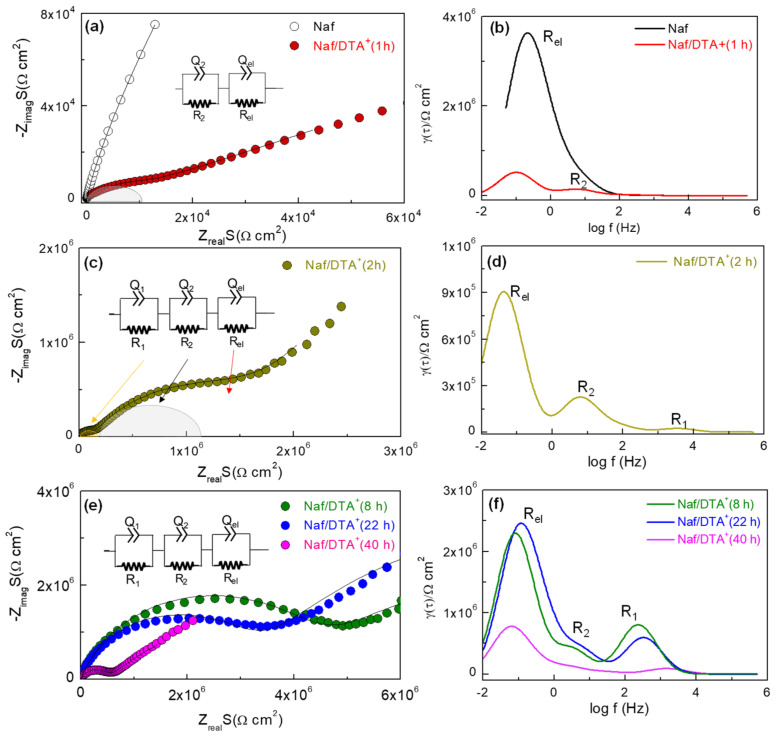
Nyquist plots of the Nafion and DTA^+^-modified Nafion films at different exchange times (**a**,**c**,**e**). The solid lines represent the fitting curve with the equivalent circuit. The corresponding DRT curves with the different processes are shown on the right (**b**,**d**,**f**).

Figure 3a,c,e shows the evolution of the Nyquist plots for the Nafion and the modified Nafion/DTA^+^ (t) films at various exchange times, where different depressed semicircles, depending on the exchange time, are observed. To better identify the different contributions in the Nyquist plots, the Distribution of Relaxation Time (DRT) method was used to deconvolute them (Figure 3b,d,f). As can be observed, the untreated Nafion film only shows a broad peak at the lowest frequency (0.1 Hz), which is assigned to the electrode polarization response, R_el_, (Figure 3a). Thus, the Nafion film has a nearly pure resistance, which was determined by observing the high-frequency intercept with the real axis. In contrast, the Naf/DTA^+^ (t) films show additional contributions at higher frequencies, denoted as R_1_ and R_2_, which are attributed to bulk and interfacial processes, respectively. It was observed that the electrode polarization response, R_el_, appears at approximately the same frequency ~10^−1^ Hz for all the films. Interestingly, the resistance of this process decreases as the immersion time in the DTACl aqueous solution increases.

The Nyquist plots for the DTA^+^-modified films were adequately fitted by using equivalent circuit models that consist of serial (RQ) elements. The following parameters were obtained for each impedance contribution: the resistance *R_i_*, the pseudocapacitance *Q_i_* and the exponential parameter *p_i_*. The real capacitance *C_i_* was determined by the following relation [41]:

(1) $$C_{i}=\frac{{\left(R_{i}Q_{i}\right)}^{1/p_{i}}}{R}$$

These data enable the estimation of the electrical resistance and capacitance values of the different resistive contributions of the films. In general, the capacitance of the high-frequency semicircle, *R*_1_, remains almost constant for all films, at about 2 × 10^−10^ F; this is a typical value for bulk ionic conduction in the films [43].

The introduction of DTA^+^ into the Nafion structure increases the resistance of the bulk conduction. This is due to the lower electrical mobility of DTA^+^ compared to protons. This effect is clearly visible in the DRT curves, where the area under the peaks is proportional to the resistance of each contribution. As shown, the resistance of R_1_ increases until t = 8 h due to the loss of protons associated with DTA^+^ inclusion. However, the resistance decreases for prolonged immersion times, which is attributed to the progressive formation of percolation paths for the DTA^+^ conduction inside the film. Similar results have already been observed for Nafion-112 and regenerated cellulose films modified with different IL-cations (BMIM^+^, OMIM^+^, DTA^+^ or Aliquat^+^) [13,40].

**Figure 4:**
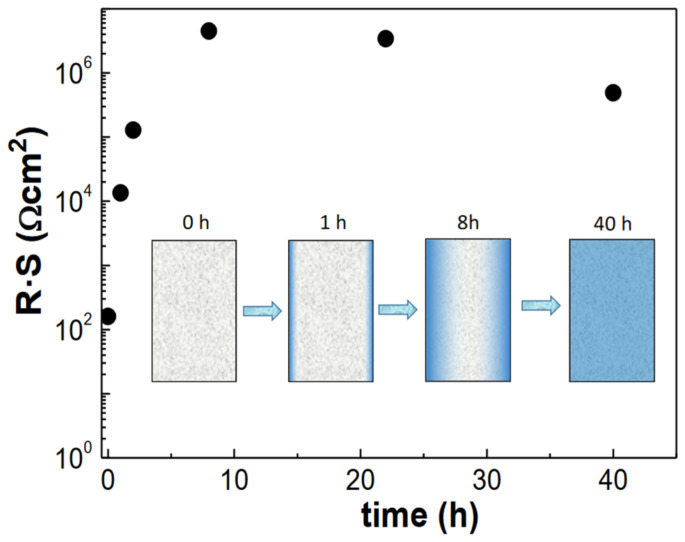
Variation in the total electrical resistance of the films with the immersion time in the DTACl aqueous solution. The inset figure shows a schematic representation of the DTA^+^ incorporation in the Nafion film over time.

The R_2_ contribution, with a capacitance of 10^−8^ F, is attributed to interfacial processes, possible due to the progressive incorporation of DTA^+^ from the film surface to the bulk. This process is initially observed for a short immersion time (1 h), increases for t = 2 h and 8 h, and then decreases for longer immersion times due to the complete exchange mechanism. Figure 4 shows the trend observed in the total electrical resistance of the films (R_1_ + R_2_), normalized by the electrode area, over the immersion time. An abrupt increase in resistance, resulting from H^+^/DTA^+^ exchange, can be observed from 160 to 4 × 10^6^ Ωcm^2^ after 8 h of immersion in the DTACl aqueous solution. Furthermore, extended immersion periods appear to enhance the formation of conductive pathways for DTA+ mobility, which results in a decrease in the overall resistance of the films.

**Figure 5:**
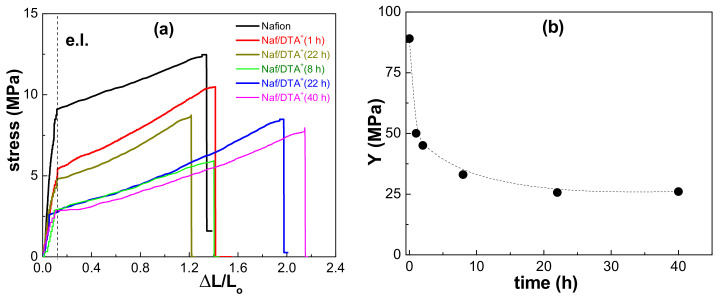
(**a**) Stress–elongation curves for the analyzed films: Nafion-212, Naf/DTA^+^ (1 h), Naf/DTA^+^ (2 h), Naf/DTA^+^ (8 h), Naf/DTA^+^ (22 h), Naf/DTA^+^ (40 h). (**b**) Variation in the Young modulus of the thin films with DTA^+^–inclusion time.

The mechanical properties of Nafion are also crucial for its use in different applications, including PEMFCs. To investigate the effect of DTA^+^ inclusion on the elastic characteristics of the Nafion film, normal tensile stress (F/S) versus strain (ΔL/Lo) curves were analyzed, as shown in Figure 5a. The results suggest that modification with DTA^+^ increases the plasticity of the samples prior to reaching the breaking point, which is consistent with the findings of previous studies on polymeric films modified with different ILs [44]. However, the elastic limit (e.l.) does not appear to be significantly affected by DTA^+^ inclusion.

The Young or elastic modulus of the films was determined from the slope of the initial linear part of the curve (Figure 5b). These results indicate that the Young modulus of the Nafion film decreases by 43% after 1 h of immersion in the DTACl aqueous solution, with a further reduction of 38% when the inclusion time increases from 1 h to 8 h. After this, the Young modulus reaches an almost constant value of 25 MPa for higher time periods. It is worth noting that the curve shape of the DTA+-modified Nafion films is similar to that reported for a similar sample measured at 85 °C at 85% RH. Nevertheless, significant changes in both the elastic modulus and elastic limit are observed as a result of hydrothermal aging in that study [45].

**Figure 6:**
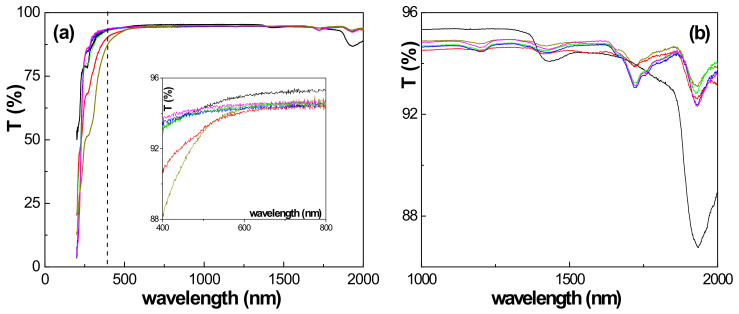
(**a**) Light transmission percentage as a function of wavelength for the analyzed films: the insert corresponds to the visible region. (**b**) Enlargement of the light transmission percentage for the near-infrared region. Nafion-212 (black line), Naf/DTA^+^ (1 h) (red line), Naf/DTA^+^ (2 h) (dark-yellow line), Naf/DTA^+^ (8 h) (green line), Naf/DTA^+^ (22 h) (blue line), and Naf/DTA^+^ (40 h) (magenta line).

Light transmission, reflection and photoluminescence are non-destructive and non-invasive contactless measurements that provide information of interest on the optical characteristics of thin films or layers deposited on a solid matrix [24,46]. In this study, wavelength dependence for light transmission curves obtained for the studied Nafion and Naf/DTA^+^ (t) films were analyzed (Figure 6). The results show the high transparency of the samples (>93%) and similar curves in the visible and near-infrared (NIR) optical regions. However, slight differences are observed at the lower wavelength interval (250 nm to 400 nm, indicated by the dashed vertical line) for Naf/DTA^+^ (1 h) and Naf/DTA^+^ (2 h). The inset in Figure 6a provides more detailed information on the effect of DTA^+^ inclusion in the Nafion film in the visible region. It shows that all the modified samples have a slightly lower light transmission (<1%) compared to the Nafion film. Additionally, the presence of DTA^+^ in the films significantly reduces the deep peak exhibited by the Nafion film at around 1930 nm, while only slightly affecting the small peak at around 1440 nm. The peaks observed at 1200 and 1720 nm are related to the presence of DTA^+^.

**Figure 7:**
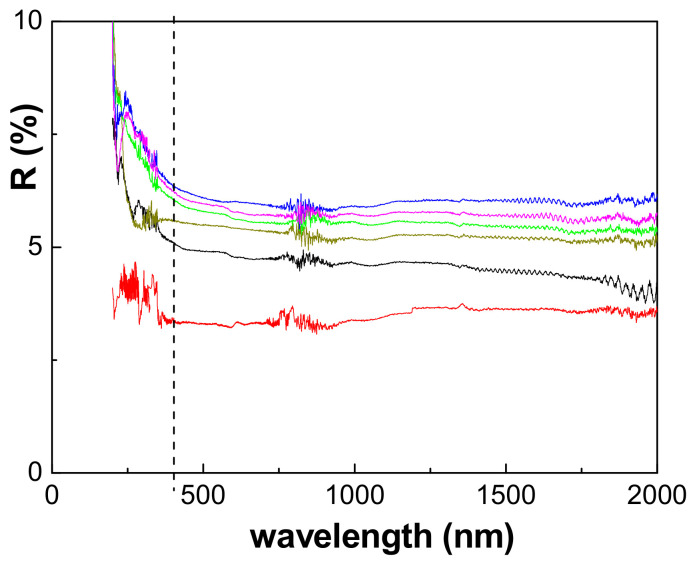
Light reflection percentage as a function of wavelength for the analyzed films: Nafion-212 (black line), Naf/DTA^+^ (1 h) (red line), Naf/DTA^+^ (2 h) (dark-yellow line), Naf/DTA^+^ (8 h) (green line), Naf/DTA^+^ (22 h) (blue line), and Naf/DTA^+^ (40 h) (magenta line).

The dependence of light reflection on the wavelength for the Nafion film and the effect of the DTA^+^ inclusion time are shown in Figure 7. The light reflection percentage remains practically constant for the wavelengths ranging between 400 nm (vertical dashed line) and 2000 nm for all DTA^+−^-modified samples, but there is a 12% reduction in the case of the untreated Nafion film. However, differences depending on the time inclusion period also exist. The inclusion of DTA^+^ in the Nafion film for 1 h reduces the reflectivity of the samples by around 25%, while different increases in the light reflection are obtained for the other Naf/DTA^+^ (t) films: 12% for Naf/DTA^+^ (2 h), 20% for Naf/DTA^+^ (8 h) and around 25% for Naf/DTA^+^ (22 h) and Naf/DTA^+^ (40 h). These percentages were determined at a wavelength of 1000 nm.

**Figure 8:**
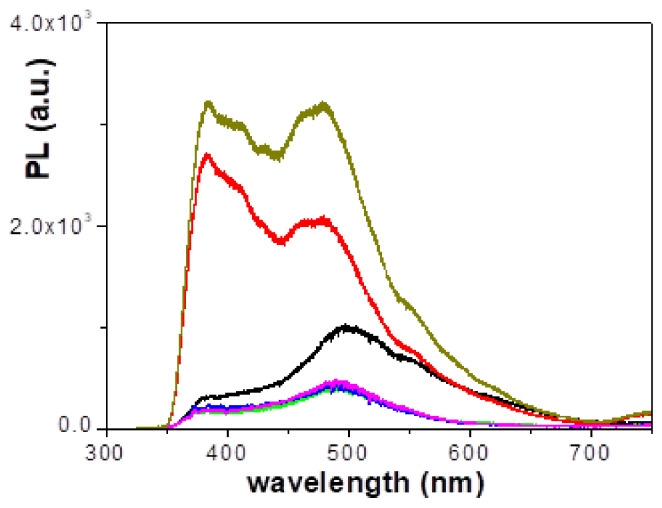
Photoluminescence spectra as a function of wavelength for the analyzed films (λ_exc_ = 325 nm): Nafion-212 (black line), Naf/DTA^+^ (1 h) (red line), Naf/DTA^+^ (2 h) (dark-yellow line), Naf/DTA^+^ (8 h) (green line), Naf/DTA^+^ (22 h) (blue line), and Naf/DTA^+^ (40 h) (magenta line).

The photoluminescence (PL) properties of Nafion films have been recently studied by Bunkin et al. [11,12]. Figure 8 shows the PL spectra obtained for the Nafion and the Naf/DTA^+^ (t) films under an excitation light of 325 nm. The Nafion film exhibits a broad shoulder at 382 nm, a peak at 498 nm and a small shoulder at 550 nm, similar to previous studies for a dry Nafion film irradiated at 369 nm (from 400 nm to 650 nm [12]). The gradual inclusion of DTA^+^ in the Nafion structure produces two opposite effects on the PL spectra, depending on the inclusion time period. For immersion periods of 1 h and 2 h in the DTACl solution, the photoluminescence intensity significantly increases (around 2–3 times for the higher peak, but 8–10 times for the broad shoulder). In contrast, for immersion periods of 8, 22 and 40 h, the PL intensity decreases (around 55 and 40% depending on the peak, for the three samples). Previous studies have already shown an increase in the PL intensity (almost 50% without curve shape change) after immersing the Nafion film in natural water [12]. On the other hand, the inclusion of DTA^+^ also slightly shifts the higher PL peak to lower wavelength values, to 480 nm for the Naf/DTA^+^ (1 h) and Naf/DTA^+^ (2 h) films, but to 492 nm for the other three samples.

The results of the optical characterizations suggest that it is possible to obtain Nafion-based films with similar light transmission but different reflection percentages, depending on the immersion time of the Nafion film in the aqueous DTACl solution. Additionally, the immersion time has a significant impact on the photoluminescence intensity of the Naf/DTA^+^ (1 h) and Naf/DTA^+^ (2 h) films. However, for prolonged immersion periods (8 ≤ t (h) ≤ 40), there is a reduction in PL intensity. These changes in the optical properties of the Naf/DTA^+^ (t) films could be of interest in various optical applications.

## 4. Conclusions

A Nafion-212 film was modified via a simple immersion process in a DTACl aqueous solution, resulting in significant changes to its physical properties. XRD, FTIR and XPS spectra analyses confirmed the incorporation of DTA^+^ into the Nafion film, leading to changes in the elemental superficial composition without altering the crystalline phases. Impedance spectroscopy data revealed an initial increase in the electrical resistance of the DTA^+^-modified films until 8 h, followed by a decrease until 40 h, indicating a complete H+/DTA+ exchange process. This finding was further supported by the DRT analysis of the different processes taking place in the modified films. In addition to the electrical properties, the inclusion of DTA^+^ affected other film characteristics, such as the elastic modulus, light reflection and photoluminescence intensity. However, the light transmission through the modified films remained similar to that of the original Nafion film, making them excellent candidates for fluorescence, sensing and fuel cell applications.

## Figures and Tables

**Table 1:** Atomic concentration (wt.%) of the different elements detected on the surface of Nafion and Naf/DTA^+^ (t) films.

Sample	F 1s (%)	C 1s (%)	O 1s (%)	S 2p (%)	N 1s (%)
Nafion-212	50.4	41.8	6.9	0.84	-
Naf/DTA^+^ (1 h)	29.5	56.8	9.7	0.95	1.0
Naf/DTA^+^ (2 h)	29.7	58.5	8.8	1.11	1.5
Naf/DTA^+^ (8 h)	36.3	49.4	8.7	1.26	1.9
Naf/DTA^+^ (22 h) *	39.0	47.2	8.2	1.14	1.6

* Analysis for Naf/DTA^+^ (40 h) could not be performed due to technical problems.

**Table 2:** Atomic concentration percentages obtained deconvoluting the different peaks obtained for the core-level spectra of carbon, oxygen and nitrogen for the Nafion and Naf/DTA^+^ (t) films.

Sample	C_A_ (%)	C_B_ (%)	C_C_ (%)	C_D_ (%)	C_E_ (%)	O_A_ (%)	O_B_ (%)	N_A_ (%)	N_B_ (%)
Nafion	24.3	9.3	1.5	3.1	3.2	0.37	0.43	-	-
Naf/DTA^+^ (1 h)	12.0	37.9	4.7	1.3	0.9	0.21	0.79	1.0	-
Naf/DTA^+^ (2 h)	11.3	36.7	6.9	1.3	1.1	0.25	0.85	1.2	0.3
Naf/DTA^+^ (8 h)	15.1	24.1	7.4	1.8	1.1	0.26	1.03	1.5	0.4
Naf/DTA^+^ (22 h)	18.3	19.6	6.3	2.0	1.0	0.24	0.76	1.0	0.6

C_A_: C–F_2_; C_B_: C–H/C–C; C_C_: C–N/C–O; C_D_: O–C=O; C_E_: C–F_3_; O_A_ = CF–O; O_B_ = SO_3_; N_A_ = C–N^+^; N_B_ = C–N.

## Data Availability

Not applicable.

## Supplementary Materials

The following supporting information can be downloaded at: https://www.mdpi.com/article/10.3390/polym15112527/s1. Figure S1: FTIR spectra of Nafion and DTA^+^- modified Nafion films at different exchange times.
