# Supplementary material for: Modification of the Physical Properties of a Nafion Film Due to Inclusion of n-Dodecyltriethylammonium Cation: Time Effect
Source: Polymers (Basel). 2023 May 30;15(11):2527. doi: 10.3390/polym15112527 (PMC10255672; doi:10.3390/polym15112527)
Supplement: Supplementary file 1 [file polymers-15-02527-s001.zip › polymers-2375201-supplementary.pdf]

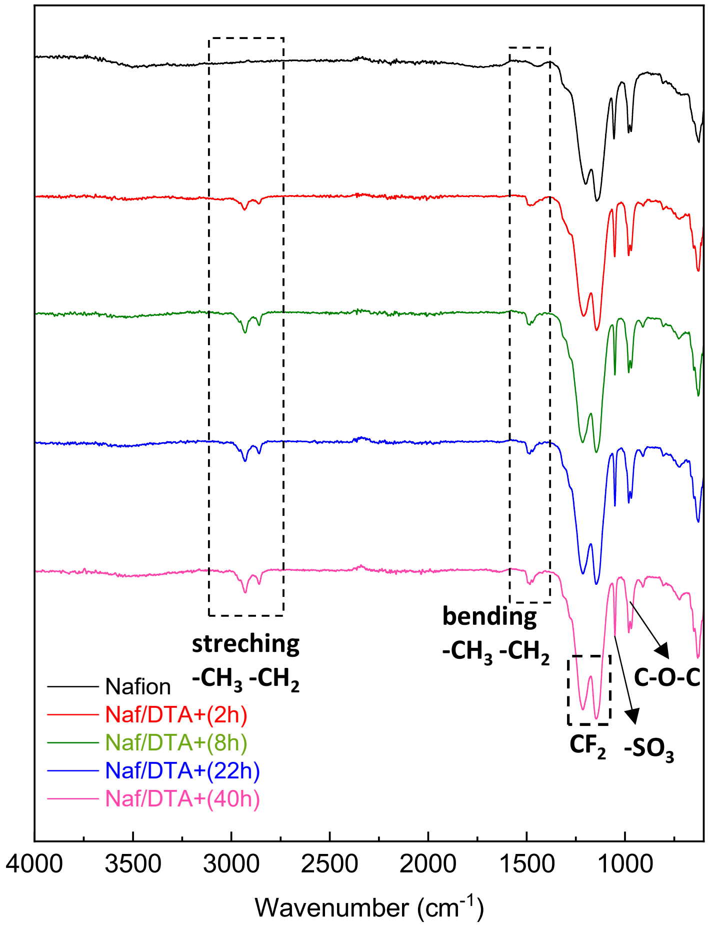

Figure S1. FTIR spectra of Nafion and DTA⁺-modified Nafion films at different exchange times.
